# Memo interacts with c-Src to control Estrogen Receptor alpha sub-cellular localization

**DOI:** 10.18632/oncotarget.10856

**Published:** 2016-07-26

**Authors:** Anna Frei, Gwen MacDonald, Ingrid Lund, Jan-Åke Gustafsson, Nancy E. Hynes, Ivan Nalvarte

**Affiliations:** ^1^ Friedrich Miescher Institute for Biomedical Research, Maulbeerstrasse, Basel, Switzerland; ^2^ Department of Biosciences and Nutrition, Karolinska Institutet, Huddinge, Sweden; ^3^ Center for Nuclear Receptors and Cell Signaling, Department of Biology and Biochemistry, University of Houston, Houston, TX, USA; ^4^ University of Basel, Basel, Switzerland

**Keywords:** Memo1, heregulin, ER alpha, estrogen, c-Src

## Abstract

Understanding the complex interaction between growth factor and steroid hormone signaling pathways in breast cancer is key to identifying suitable therapeutic strategies to avoid progression and therapy resistance. The interaction between these two pathways is of paramount importance for the development of endocrine resistance. Nevertheless, the molecular mechanisms behind their crosstalk are still largely obscure. We previously reported that Memo is a small redox-active protein that controls heregulin-mediated migration of breast cancer cells. Here we report that Memo sits at the intersection between heregulin and estrogen signaling, and that Memo controls Estrogen Receptor alpha (ERα) sub-cellular localization, phosphorylation, and function downstream of heregulin and estrogen in breast cancer cells. Memo facilitates ERα and c-Src interaction, ERα Y537 phosphorylation, and has the ability to control ERα extra-nuclear localization. Thus, we identify Memo as an important key mediator between the heregulin and estrogen signaling pathways, which affects both breast cancer cell migration and proliferation.

## INTRODUCTION

Estrogen (E2) binds and activates estrogen receptors (ERs) to enter the nucleus and regulate the expression of genes involved in cell survival, proliferation and differentiation [1]. In hormone responsive breast cancer, ER alpha (ERα) is an important driver of proliferation, and is a first-line target for therapy. However, ERα-positive breast cancers often acquire endocrine resistance and escape such therapy, likely through the activation of alternative mitotic pathways or ligand independent activation of ERα [2]. Phosphorylation of ERα can regulate both its ligand-dependent and -independent transcriptional activity. The phosphorylation of ERα at tyrosine 537 (Y537) is mediated by the c-Src (Src) kinase and promotes ERα hormone-binding, dimerization, and activity [3–6]. However, this phosphorylation is also known to increase ERα association with Src to promote Src activation, as well as ERα extra-nuclear localization [5, 7, 8].

Mediator of ErbB2-driven cell motility, Memo (gene name *MEMO1*), is a small ubiquitous redox-active protein with an important role in breast cancer cell migration, invasion, and metastasis downstream of growth factor signaling [9, 10]. Memo is located both in the nucleus and cytoplasm [11, 12], and its expression has been found to be increased in >40% of primary breast tumors, where its cytoplasmic localization correlated with aggressive disease parameters, such as Luminal B subtype, early-distant recurrence, and death [9]. Apart from conveying migratory signals from receptor tyrosine kinases (RTKs) such as the human epidermal growth factor receptor 2 (HER2), Memo was recently shown to interact with ERα and ERβ and to promote ERα phosphorylation and ligand-independent activation [13]. These data suggest that in addition to RTK signaling Memo may have an important role in estrogen signaling. However, how Memo might function to integrate these two mitogenic pathways has not been explored. Memo is known to have a role in heregulin- (HRG) mediated migration [9, 10] in breast cancer cells. Here, we provide mechanistic insight into how Memo controls ERα function and E2-mediated cell migration. We show that Memo regulates ERα function through controlling its extra-nuclear localization. This is especially striking upon simultaneous activation of the HRG and E2 pathways. Further, Memo promotes the interaction between ERα and Src in the cytoplasm and increases Src Y418 and ERα Y537 phosphorylation with consequences for proliferation and migration, as well as endocrine treatment response. Based on these results, we propose that Memo resides at the intersection between HRG and E2 signaling in breast cancer, and is an important new player in the crosstalk between the two pathways.

## RESULTS

### Memo controls HRG-mediated expression of ERα target genes

**Figure 1:**
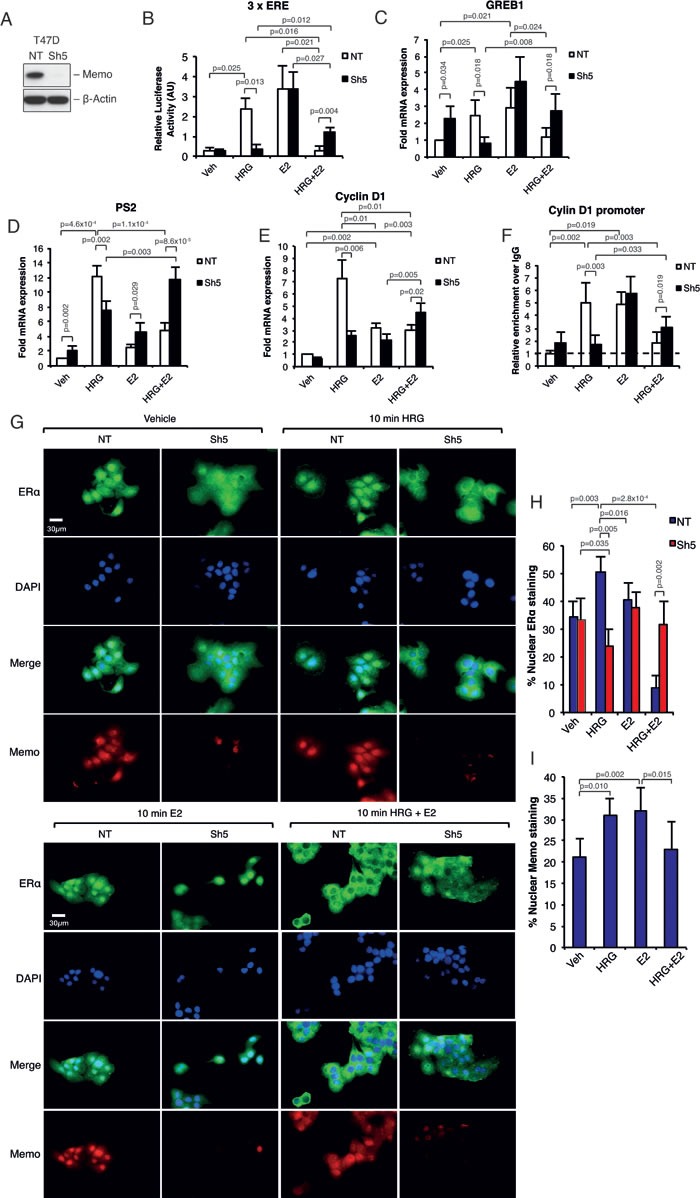
Memo controls HRG-mediated ERα target gene expression and ERα extra-nuclear localization **A.** Western blot analysis of Memo protein levels in T47D NT control and Sh5 Memo KD cells. **B.** ERα activity upon E2 and or HRG stimulation for 48h. Firefly luciferase activity was normalized to renilla luciferase activity (n = 3). **C.** Relative mRNA expression of GREB1 in T47D cells cultured in the presence of DMSO (Vehicle, Veh), HRG, E2 and HRG+E2 for 24 h (n = 5). **D.** Relative mRNA expression of PS2 in T47D cells cultured as in (B) (n = 5). **E.** Relative mRNA expression of Cyclin D1 in T47D cells cultured as in (B) but for 6h (n = 3). **F.** ChIP analysis showing the recruitment of ERα to the ERE promoter sequence of Cyclin D1 in T47D NT and Sh5 cells treated with DMSO, HRG, and/or E2 for 30 min (n = 3). Dotted line indicates enrichment level with non-specific IgG. **G.** Immunofluorescence (IF) analysis showing the cytoplasmic/nuclear localization of ERα and Memo in NT and Sh5 T47D cells treated with DMSO (vehicle), HRG and/or E2 for 10 minutes. Nuclei were stained with DAPI. 40x magnification, Scale bar: 30 μM. **H.** Quantification of nuclear ERα IF intensity as percentage of total ERα IF intensity (n = 7). **I.** Quantification of nuclear Memo IF intensity as percentage of total Memo IF intensity (n = 5). The data shown in (B-F) and (H-I) represent means and error bars represent standard deviation (S.D.), *P* values were determined using Student's t-test or one-way ANOVA.

To analyze the function of Memo on ERα downstream of E2 and HRG we chose the ERα+ / HER2+ (non-overexpressing, luminal B-like) human breast cancer cell line T47D stably expressing a control, non-targeting (NT), short hairpin RNA (shRNA), or one expressing Memo shRNA (Sh5) (Figure 1A). When analyzing the ERα transcriptional activity in these cells by using a 3 x ERE luciferase reporter construct, there was no difference in E2 mediated ERα activity upon Memo knockdown (Sh5) (Figure 1B). As described before [14], treating the cells with HRG significantly increased ERα activity, however, only in control NT cells. Surprisingly, combined E2 and HRG treatment decreased ERα's activity compared to E2 treatment for NT cells, and to a lesser extent for Sh5 cells (Figure 1B). This was also reflected in the expression of the classical ERα target genes GREB1 (Figure 1C), PS2 (Figure 1D) and Cyclin D1 (Figure 1E). We did not see any decrease in their E2-mediated expression upon Memo knockdown. In contrast, we found a slight, and sometimes significant, increase in their basal (E2-independent, Figure 1C, 1D) and E2-mediated (Figure 1D) expression in Sh5 cells. Interestingly, combined HRG and E2 treatment generally increased expression more in Sh5 cells compared to NT cells. Although E2 treatment only gave a ~2.5 fold increase in Cyclin D1 expression (Figure 1E), we could detect a significant enrichment of ERα at the estrogen response element (ERE) promoter site of this gene (Figure 1F). The HRG treatment also increased ERα enrichment in NT but not in Sh5 cells. In contrast, combined HRG and E2 treatment increased ERα binding only in Sh5 cells (Figure 1F). This implies that ERα can increase cyclin D1 mRNA expression by its enrichment at the promoter of cyclin D1 upon HRG and E2 stimulation. These data also suggest that Memo promotes HRG-mediated ERα activation and promotes antagonizing effects of HRG on E2-induced ERα activity.

### Memo regulates ERα nuclear localization downstream of HRG

Lower nuclear levels or lower general levels of ERα could account for the observed differences in ERα target gene expression between NT and Sh5 cells. However, there were no apparent differences in ERα mRNA or protein levels between NT and Sh5 cells (Supplementary Figures 1A and 2). In contrast, we saw a striking difference in ERα nuclear localization upon HRG and combined HRG and E2 treatment. HRG treatment caused an increased ERα nuclear localization in NT cells already following 10 min HRG treatment, which was absent in Sh5 cells (Figure 1G and 1H; Supplementary Figure 1B). Combined HRG and E2 treatment almost totally abolished ERα nuclear localization in NT cells, while its localization in Sh5 cells was not affected (Figure 1G and 1H; Supplementary Figure 1B). After 45 min of treatment ERα relocalized to its original localizations in both NT and Sh5 cells (Supplementary Figure 1B). Furthermore, Memo localized to the nucleus upon HRG or E2 treatment (Figure 1G and 1I; Supplementary Figure 1C). We also observed similar effects after stimulation with 10 min HRG and E2 on the nuclear localizations of ERα and Memo in parental MCF-7 cells (Supplementary figure 2A, B), suggesting that our observations could be a general phenomenon for HRG-responding ERα-positive breast cancer cells. Further, HRG treatment also promoted Memo to localize to membrane ruffles in T47D cells [11, 15] and co-localized with ERα at these sites (Supplementary Figure 1D).

### Memo promotes ERα and c-Src phosphorylation

**Figure 2:**
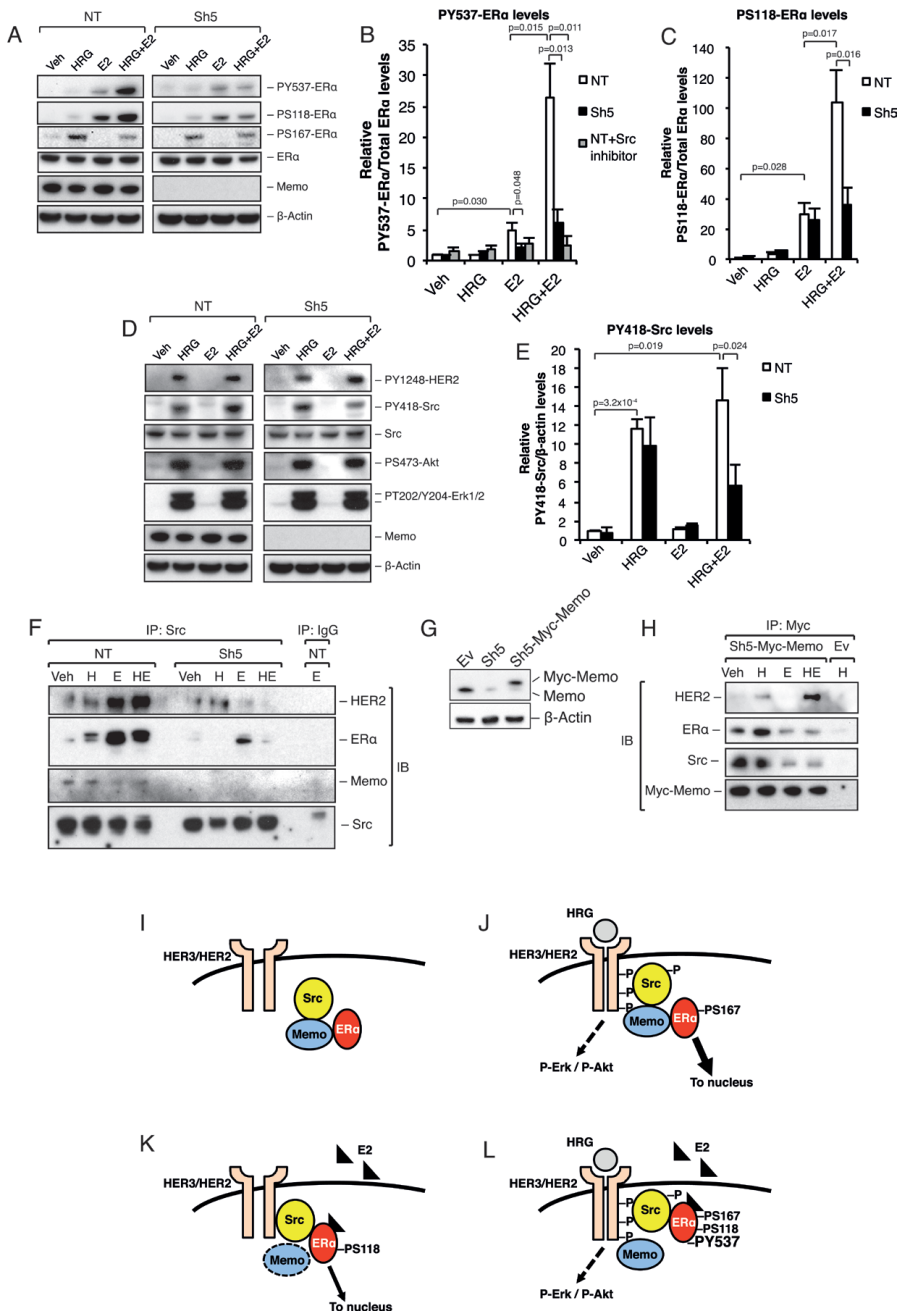
Memo promotes ERα phosphorylation and interaction with Src upon HRG and E2 treatment **A.** Western blot analysis of ERα phosphorylation status in T47D NT and Sh5 cells treated with DMSO (Veh), HRG, and/or E2 for 10 min. **B.** Quantification of relative PY537-ERα levels (n = 3) in the presence or absence of 500 nM Src inhibitor-1. **C.** Quantification of relative PS118-ERα levels (n = 3). **D.** Western blot analysis of HER2, Src, Akt, and Erk1/2 phosphorylation status in T47D NT and Sh5 cells treated with DMSO, HRG, and/or E2 for 10 min. **E.** Quantification of relative PY418-Src levels (n = 3). **F.** Immunoprecipitation (IP) of Src in NT and Sh5 T47D cells treated for 10 min with DMSO (Veh), HRG (H) and/or E2 (E), followed by immunoblotting (IB) for HER2, ERα, Memo and Src. **G.** Memo and Myc-Memo protein levels in T47D cells transfected with an empty pLHCX vector (Ev), Sh5 KD construct, and Sh5 cells transfected with pLHCX-Myc-Memo (Sh5-Myc-Memo). **H.** IP of Myc-Memo in T47D Sh5-Myc-Memo cells treated for 10 min with DMSO (Veh), HRG and/or E2, followed by immunoblotting (IB) for HER2, ERα, Src, and Memo. T47D Ev cells were used as a control. **I.** Proposed model for Src, Memo and ERα interaction (I-L). Under basal conditions, without HRG or E2 stimulation, Memo associates with both Src and ERα. However, Src and ERα do not appear to bind each other directly. **J.** HRG treatment induces HER2 heterodimerization and phosphorylation as well as recruitment of the Memo-Src-ERα complex to HER2. The HER2 activation promotes phosphorylation and activation of Src as well as Erk1/2 and Akt pathways. This in turn promotes ligand independent activation, likely through its phosphorylation at S167, and a strong Memo-dependent ERα nuclear translocation. **K.** Upon E2 treatment ERα changes conformation that likely disrupts the ERα-Memo interaction. However, the ERα interaction with the Src-HER2 complex is still Memo dependent. E2 promotes ERα S118 and Y537 phosphorylation, resulting in ERα activation and nuclear translocation. **L.** Upon combined HRG and E2 treatment, Memo binds to HER2, and the E2 binding to ERα prevents Memo's complex formation with ERα. Nevertheless, Memo is required for this Src-ERα interaction. The HRG activation of HER2 and Src increases the binding of ERα to Src. This in turn increases the phosphorylation of ERα on S118 and especially Y537, resulting in a very tight Src-ERα complex and preventing ERα from entering the nucleus. The data shown in (B, C, E) represent means and error bars represent standard deviation (S.D.). The IPs (F) and (H) are representative of 3 independent experiments. *P* values were determined using Student's t-test or one-way ANOVA.

Previous reports [5, 7, 8] have described the Src-dependent phosphorylation of ERα at Y537 (PY537-ERα) as being required for its extra-nuclear localization. Interestingly, we could already observe a strong increase in PY537 and PS118-ERα levels after 5-10 min of combined HRG and E2 treatment (Figure 2A-2C; Supplementary Figure 3A-3B). This increase was significantly lower in Sh5 cells. No clear difference was observed for PS167-ERα (Figure 2A). In addition, PY418-Src was significantly increased in NT cells compared to Sh5 cells, however, only upon combined HRG and E2 treatment (Figure 2D, 2E). We could not observe any significant difference in Y1248-HER2, S473-Akt, and T202/Y204-Erk1/2 phosphorylation between the NT and Sh5 cells (Figure 2D). In order to test the dependence of ERα-Y537 phosphorylation on active Src we used a Src inhibitor. Src inhibition significantly lowered ERα-Y537 phosphorylation to levels comparable to those in Sh5 cells (Figure 2B, gray bars, Supplemental Figure 3C). Our data suggest that the difference in ERα sub-cellular localization between NT and Sh5 cells stimulated with HRG and E2 may be due to differences in PY537-ERα levels mediated to a significant extent by Src and Memo.

### Memo interacts with Src and promotes its interaction with ERα

ERα phosphorylation at Y537 is known to promote a tight extra-nuclear interaction with Src, which increases PY418-Src levels and Src activation [5, 7, 8]. We hypothesized that such an interaction could underlie ERα extra-nuclear localization upon combined HRG and E2 treatment. Interestingly, immunoprecipitation (IP) of cytoplasmic Src revealed enriched binding to ERα upon E2 and combined E2-HRG treatment (Figure 2F). This interaction was greatly reduced in Sh5 cells. In addition, HER2 also interacted with Src upon E2 and combined E2-HRG treatment. Interestingly, the opposite was seen for Memo that interacted more with Src upon basal conditions or upon treatment with only HRG (Figure 2F).

To further study the interaction between Memo and the Src-ERα complex we used T47D Sh5 cells stably expressing a Myc-tagged Memo rescue vector (Figure 2G). We observed that Myc-tagged Memo interacted with Src and ERα under basal conditions and upon HRG treatment (Figure 2H). Treatments with E2 decreased this interaction. Memo also interacted with HER2, however, only upon HRG and combined HRG-E2 treatment (Figure 2H). These data are summarized in Figure 2I-2L, and suggest that Memo is necessary for the interaction between ERα, Src, and HER2. These data also demonstrate that this interaction is not necessarily dependent on an active Src.

### ERα Y537F mutant inhibits ERα extra-nuclear localization similar to Memo knockdown

**Figure 3:**
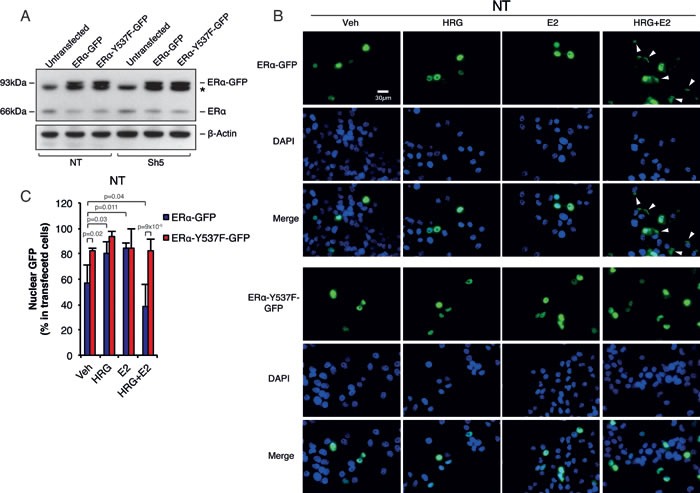
Memo controls ERα extra-nuclear localization through ERα-Y537 phosphorylation **A.** Western blot analysis of endogenous ERα and GFP-tagged WT or Y537F ERα. Asterisk indicates an unspecific band. **B.** Immunofluorescence (IF) analysis showing the cytoplasmic/nuclear localization of ERα-WT-GFP and ERα-Y537F-GFP in NT T47D cells treated with DMSO (vehicle), HRG and/or E2 for 10 minutes. Nuclei were stained with DAPI. 40x magnification, Scale bar: 30 μM. **C.** Quantification of nuclear ERα-GFP IF intensity as percentage of total ERα IF intensity (n = 6). The data shown in (C) represent means and error bars represent standard deviation (S.D.), *P* values were determined using one-way ANOVA.

In order to confirm that Memo mediates its effect on ERα extra-nuclear localization through the ERα-Y537 phosphorylation site, we generated an ERα-Y537F mutant. It has previously been shown that the ERα-Y537 site is needed for ERα nuclear export [7]. Overexpression of ERα-WT-GFP and ERα-Y537F-GFP constructs in T47D NT and Sh5 cells (Figure 3A) resulted in generally higher nuclear ERα-WT-GFP levels under basal conditions (~55% nuclear GFP) (Figure 3B, 3C, and Supplementary Figure 4A, B) compared with endogenous ERα (~35%) (Figure 1G, 1H). Treatment of NT cells with HRG or E2 resulted further increased ERα-WT-GFP nuclear localization (~80%). However, similar to endogenous ERα, combined HRG and E2 treatment greatly reduced the nuclear ERα-WT-GFP levels (to ~40%) (Figure 3B, 3C). The ERα-Y537F-GFP mutant overexpressing cells had high basal nuclear ERα levels and these levels did not change with any treatment combination (Figure 3B, 3C). In contrast to NT cells, overexpression of ERα-WT-GFP in Sh5 cells did not result in increased nuclear localization upon HRG treatment, and no decreased nuclear localization could be observed upon combined HRG+E2 treatment (Supplementary Figure 4A-C). These data suggest the Memo can control ERα extra-nuclear localization via ERα-Y537 phosphorylation downstream of HRG and E2.

### The effects of Memo on ERα requires a functional ERα

**Figure 4:**
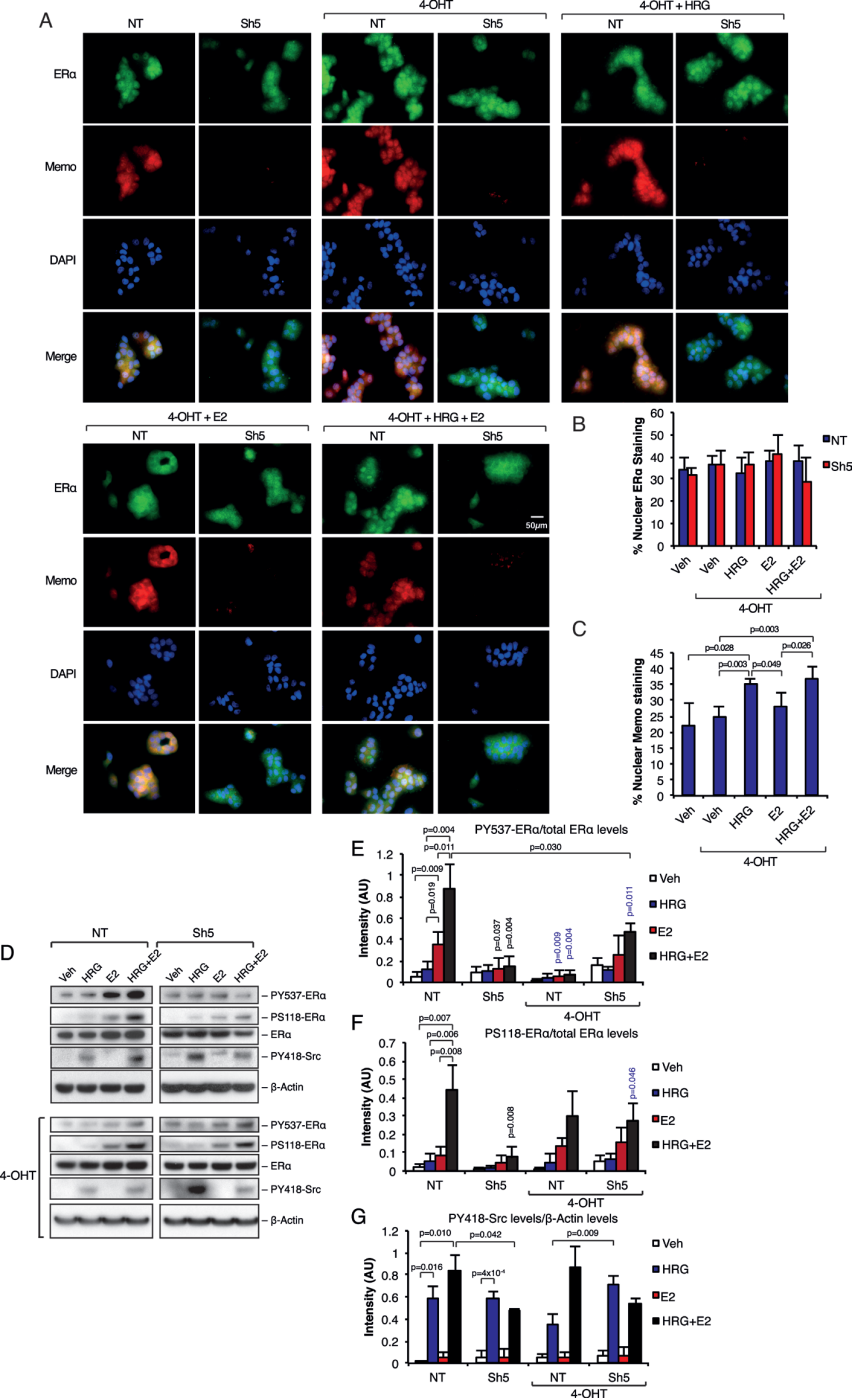
Inhibition of ERα abolishes the effects of Memo on ERα **A.** Immunofluorescence (IF) analysis showing the cytoplasmic/nuclear localization of ERα and Memo in NT and Sh5 T47D cells treated with 4-OHT, HRG and E2 for 10 minutes. Nuclei were stained with DAPI. 40x magnification, Scale bar: 50 μM. **B.** Quantification of nuclear ERα IF intensity as percentage of total ERα IF intensity (n = 4). **C.** Quantification of nuclear Memo IF intensity as percentage of total Memo IF intensity (n = 4). **D.** Western blot analysis of PY537-ERα, PS118-ERα, and PY418-Src levels in starved T47D cells treated with 2 nM HRG, 10 nM E2, DMSO (Veh), and/or 20 nM 4-OHT for 10 min. **E.** Quantification of PY537-ERα levels relative to total ERα levels (AU, arbitrary units) (n = 3). **F.** Quantification of PS118-ERα levels relative to total ERα levels (AU, arbitrary units) (n = 3). **G.** Quantification of PY418-Src levels relative to β-Actin levels (AU, arbitrary units) (n = 3). The data shown in (B, C, E - F) represent means and error bars represent S.D. Significance levels written in blue show the significance between 4-OHT treatment and the respective treatment without 4-OHT. *P* values were determined using Student's t-test or one-way ANOVA.

We next investigated if the Memo-mediated sub-cellular localization and phosphorylation of ERα can be blocked by the ERα antagonist 4-hydroxy tamoxifen (4-OHT). We could not observe any difference in ERα sub-cellular localization when the cells were treated with 4-OHT in combination with HRG and/or E2 (Figure 4A and 4B), which was in contrast to the effects seen in Figure 1 (Figure 1G and 1H). On the other hand, Memo localized to the nucleus upon the HRG treatments in the presence of 4-OHT (Figure 4A and 4C). Furthermore, 4-OHT abolished the PY537-ERα in T47D NT cells in response to E2 and HRG+E2 (Figure 4D and 4E). Surprisingly, 4-OHT treatment slightly, but significantly increased PY537-ERα in Sh5 cells in response to combined HRG and E2 stimulation. On the other hand, there was no difference in PS118-ERα between NT and Sh5 cells upon combined HRG and E2 stimulation with concomitant 4-OHT treatment (Figure 4D and 4F). This suggests that, in contrast to PY537-ERα, the effect of 4-OHT on PS118-ERα is independent of Memo. 4-OHT treatment did not give any significant difference in PY418-Src phosphorylation compared to 4-OHT untreated cells (Figure 4D and 4G). However, PY418-Src was significantly higher in Sh5 cells upon combined 4-OHT and HRG treatment compared to similarly treated NT cells (Figure 4G, blue bars). These phosphorylation patterns were also observed using T47D Sh1 cells with less effective Memo knockdown (Supplementary Figure 5A-5E). Our data suggest that ligand-activated ERα is needed for the effect of Memo on ERα sub-cellular localization and phosphorylation.

### Memo mediates migratory and proliferation effects downstream of HRG and E2

**Figure 5:**
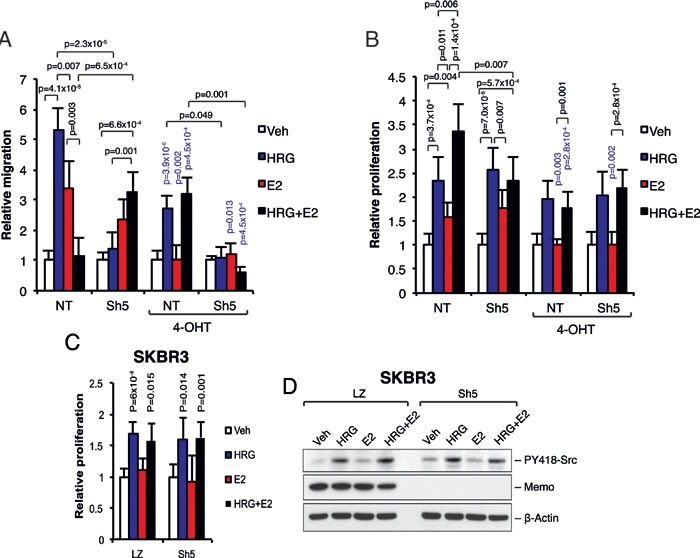
Memo together with ERα regulates cell migration and proliferation downstream of HRG and E2 **A.** Migration assay. Starved T47D cells were seeded into the upper transwell chamber. The lower wells contained phenol-free DMEM supplemented with 0.5% DCC-treated FCS and 2 nM HRG, 10 nM E2, DMSO (Veh), and/or 20 nM 4-OHT. After 24 h the migrated cells were fixed, stained and counted. The migration is expressed relative to respective DMSO treated cells (n = 5). **B.** Proliferation assay. T47D cells were starved for 5 days in phenol-free DMEM supplemented with 0.5% DCC-treated FCS, and in the presence of 2 nM HRG, 10 nM E2, DMSO (Veh), and/or 20 nM 4-OHT. Viable cells were counted and the proliferation change was assessed relative to respective DMSO treated cells (n = 5). **C.** Proliferation of LZ (control) and Sh5 (Memo KD) SKBR3 cells was assessed as in (B) in the presence of 2 nM HRG, 10 nM E2, and/or DMSO (Veh). Viable cells were counted and the proliferation change was assessed relative to respective DMSO treated cells (n = 5). **D.** Western blot analysis of PY418-Src levels in LZ and Sh5 SKBR3 cells treated with 2 nM HRG, 10 nM E2, and/or DMSO (Veh) for 10 min. The data shown in (A - C) represent means and error bars represent S.E.M. The data shown in (D) is representative of 3 independent experiments (n = 3). Significance levels written in blue show the significance between 4-OHT treatment and the respective treatment without 4-OHT. *P* values were determined using Student's t-test or one-way ANOVA.

Finally, we analyzed the effect of Memo on migration and proliferation downstream of HRG and E2. As described before [9, 10], we could observe Memo-dependent cell migration upon 24h HRG treatment (Figure 5A). Treating the cells with E2 induced cell migration in both NT and Sh5 cells. Interestingly, combined HRG and E2 treatment did not induce cell migration in NT cells but did so in Sh5 cells, which migrated similarly to when stimulated by E2 alone. Treating the cells with 4-OHT decreased migration in all cases with the exception of NT cells treated with combined HRG+E2, where migration was induced to similar levels as seen for HRG treatment (Figure 5A). Similar effects on migration were observed using T47D Sh1 cells that have a less effective Memo KD (Supplementary Figure 6A).

It was previously reported that Memo is required for E2-dependent and independent MCF-7 cell growth [13]. In our T47D model we could see an increase in proliferation with HRG and E2 treatments, however, this increase was similar between the NT and Sh5 cells (Figure 5B). In contrast, combined HRG and E2 treatment increased proliferation of NT cells compared to Sh5 cells (Figure 5B). Treating the cells with 4-OHT totally abolished the E2-mediated proliferation and slightly reduced the HRG-mediated proliferation in both NT and Sh5 cells (Figure 5B). Similar effects on proliferation were observed using the less effective Memo knockdown T47D Sh1 cells (Supplementary Figure 6B). Furthermore, the increase in proliferation of NT cells upon combined HRG+E2 treatment could not be observed in NT cells overexpressing the ERα-Y537F mutant, but instead proliferation of these cells was comparable to Sh5 cells (Supplemental Figure 6C), suggesting that the ERα-Y537 phosphorylation is essential for the Memo-dependent proliferation downstream of combined HRG and E2 stimulation.

To elucidate the dependence of ERα on Memo-mediated proliferation, we used the HER2+/ERα- breast cancer cell line SKBR3 treated with E2 and/or HRG. Here we could not observe any difference in proliferation with or without Memo (LZ control versus Sh5 Memo KD) and with or without E2 and HRG treatment combinations (Figure 5C). Interestingly, in contrast to T47D cells, the SKBR3 cells did not show any obvious reduction in PY418-Src upon combined HRG and E2 treatment (Figure 5D versus Figure 2D and 2E). These results suggest that Memo together with a ligand-activated ERα is needed for the combined effect of HRG and E2 on cell migration and proliferation, and that this is possibly mediated by Src phosphorylation and activation.

In summary, we show that Memo controls cell migration and promotes proliferation downstream of simultaneous HRG and E2 stimuli, and that this is likely mediated by a regulatory role of Memo in the ERα-Src interaction, their phosphorylation and their function.

## DISCUSSION

Endocrine therapy resistance resulting in metastatic recurrence contributes to death of many breast cancer patients. Memo plays an essential role in breast cancer metastasis [9] and in this study we show that Memo has a novel role at the hub between E2 and growth factor signaling, mediating crosstalk between these pathways. Our data suggest that Memo determines the localization, phosphorylation, and thus the function of ERα downstream of the HER2 receptor, and that this is achieved through a Memo-dependent interaction of ERα with Src. We show that Memo has a regulatory role on cell migration and proliferation downstream of a combined HRG and E2 stimulus in the ERα and HER2-positive breast cancer cell line T47D, and that this has consequences for tamoxifen treatment.

It is known that ERα interacts with Src and that this affects phosphorylation and activation of both [3–6]. Here we show that this interaction is dependent on a single protein, Memo, making it an important player in the cross-regulation of the HRG and E2 pathways in breast cancer.

**Figure 6:**
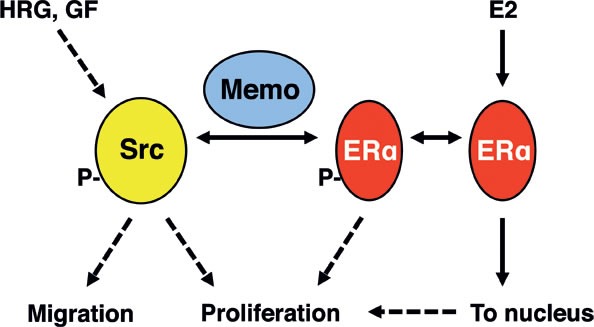
Summary model Src is activated by various growth factor (GF) receptors, but also by E2 stimulation, at least in the presence of GFs. Memo interacts with Src and ERα to promote their binding upon HRG and E2 stimulation. This in turn increases Y418-Src and Y537-ERα phosphorylation as well as the extra-nuclear retention of ERα. Memo is thus a crucial component at the intersection between E2 and HRG (and possibly other GF) signaling, and can thus promote their downstream effect on cell migration and proliferation.

About 30% of breast cancer patients express HRG, often in the absence of the *ERBB2* amplicon [16, 17] of which many are both HER2 and ERα-positive (e.g. Luminal B subtype). Memo was found to be expressed in >40% of a cohort of primary breast tumors [9] and was localized to both the cytoplasm and the nucleus. Interestingly, its extra-nuclear localization correlated with aggressive molecular disease parameters (such as high grade, ERα/PR-negative, HER2-positive), as well as triple-negativity and Luminal B subtypes. Inversely, high nuclear Memo was associated with good prognostic factors, such as low grade and ERα/PR positivity [9]. In the context of HER2 and ERα-positive breast cancer, our data show that both Memo and ERα can rapidly co-localize to the nucleus upon HRG or E2 stimulation. The nuclear function of Memo here is still unknown. In contrast, upon simultaneous HRG and E2 stimulus both Memo and ERα are mainly localized extra-nuclear, thus preventing ERα transcriptional activity. Antagonizing effects of HRG on E2 signaling have previously been described [18–21]. Here we show that this can be mediated by Memo, which is able to increase the ERα-Src interaction, resulting in elevated PY537-ERα and PY418-Src, and a complex unable to enter the nucleus. In fact, these phosphorylations are needed for a tight interaction between extra-nuclear ERα-Src [5, 7]. This may lead to the lower ERα transcriptional activity (Figures 1B-1F), but increased proliferation (Figure 6B) that we observed following HRG+E2 addition to cells. Although it is possible that increased Src activation contributes to this finding, the exact mechanism governing the rapid ERα extra-nuclear localization upon HRG and E2 treatment, as well as the potential for other factors to contribute to the ERα-Src interaction, is not fully understood. Efforts here could lead to better understanding and new treatment of breast cancer, in particular since this state would mimic the situation of premenopausal patients where circulating E2 and growth factors are present. Our data suggest that these patients may have lower nuclear Memo (and ERα) levels, which would correlate with aggressive disease parameters [9]. Interestingly, the increased proliferation upon combined HRG+E2 treatment in NT cells (Figure 6B) was inhibited by 4-OHT, resulting in proliferation similar to Sh5 cells. These results lead us to propose that Memo might be diagnostic and indicative of a better response to endocrine treatment. On the other hand, Memo may be responsible for increased cell migration and metastasis upon inhibiting ERα with 4-OHT. This is intriguing and deserves deeper characterization since such knowledge may be of importance in understanding how tumor cells escape the primary site and if 4-OHT may promote this effect in a Memo-dependent manner in ERα-positive cells. Our data also suggest that ERα and Memo may influence the activity of each other with ensuing effects on cell migration (Figure 5A).

Memo is a copper-dependent redox protein that can change the oxidation status and function of RhoA as well as the activity of NADPH-oxidases [9]. Although beyond the scope of this report, the function of Memo's redox activity in the context of ERα signaling will be very interesting to explore in the future, especially since it may have the potential to be blocked by small molecule ligands. In this respect, future studies with xenograft models would be interesting. However, the lack of metastasizing ER+/HER2+ breast cancer models currently prevent such studies. In summary, we propose that Memo connects E2 and HRG signaling through its interactions with ERα and Src. Thus, Memo functions as a molecular hub in relaying E2 and growth factor signaling towards proliferation and migratory outputs (Figure 6). This is of relevance since breast cancer is regulated by hormonal and/or growth factor cues. In addition, the frontline treatment with 4-OHT of ER+ breast cancer may have different consequences if Memo is overexpressed or not, or whether it is localized to the nucleus or cytoplasm. Our data may be of value in understanding and treating breast cancer.

## MATERIALS AND METHODS

### Cells and culturing

The human breast cancer cell lines T47D, MCF-7, and SKBR3 (all authenticated by vendor, ATCC, Wesel, Germany) were cultured in Dulbecco's modified Eagle's medium (DMEM) (containing 4.5 mg/ml glucose) supplemented with 10% Fetal calf serum, FCS (all from ThermoFisher Scientific, Stockholm, Sweden) in 5% CO2 at 37°C in a humidified incubator. Prior to treatment with 10 nM estradiol (E2), 2 nM Heregulin-1β (HRG) (both from Sigma Aldrich, St Louis, MO, USA), 20 nM 4-hydroxytamoxifen (4-OHT, Tocris, Bristol, UK), and/or 500 nM Src inhibitor-1 (Sigma Aldrich) the cells were starved for 48h in phenol-free DMEM containing 0.5% Dextran-charcoal (DCC) treated FCS. The generation of stable NT, LZ, Sh5, Sh1, and pLHCX-Myc-Memo cell lines was described before [9]. All cell lines were confirmed to be mycoplasma free.

### Real-time qPCR analysis

Total RNA was isolated from cells using QIAshredder and RNeasy Mini Kit (Qiagen, Hombrechtikon, Switaerland). cDNA synthesis and amplification was performed using the Superscript III first-strand synthesis kit according to manufacturer's instructions (ThermoFisher). Quantitative real-time PCR (qPCR) analysis was performed with the Fast SYBR Green Master Mix (ThermoFisher) and the ABI7500 real-time PCR (ThermoFisher). Data was normalized to 18S rRNA. See Supplemental Table I for primer sequences.

### Western blot analysis and immunoprecipitation

Total cell extracts were prepared by resuspending the harvested cells in RIPA lysis buffer (50 mM TrisHCl, pH 8.0, 150 mM NaCl, 5 mM EDTA, 0.5% (v/v) Nonidet P-40, 0.5% (v/v) Triton X-100, 0.1% Sodium Deoxycholate, 2 mM Na_3_VO_4_, 1 mM DTT, and 1x EDTA-free protease inhibitor cocktail (Roche, Basel, Switzerland)). The extracts were incubated 30 min on ice and then centrifuged at 10 000 x g, 10 min at +4°C. Protein concentration was measured and 30 μg was separated on a 4 - 12% SDS-PAGE gel. The proteins were then transferred to a PVDF membrane (Bio-Rad laboratories, Hercules, CA, USA) and blocked using StartingBlock T20 buffer (Thermo Fisher). The membrane was probed using the primary and secondary antibodies listed in Supplemental Table II, followed by signal detection using ECL Prime kit and light sensitive films (GE Healthcare, Uppsala, Sweden). Quantification was performed using the ImageJ software (NIH Software, Bethesda, MD, USA). For immunoprecipitations, 400 μg cytoplasmic lysate was incubated over-night with 5 μg of Src antibody (N-16, Sc-284, Santa Cruz, Dallas, TX, USA) coupled to Dynabeads Protein G magnetic beads (ThermoFisher), or with 40 μl anti-c-Myc magnetic beads (ThermoFisher). 5 μg of non-targeting rabbit IgG (sc-2763, Santa Cruz) was used a control where appropriate. The bound complex was eluted using 100 mM Glycine pH 2.8 or with 50 μg purified c-Myc peptide (ThermoFisher), and resolved on SDS-PAGE and blotted as described above. Elution with the c-Myc peptide is a prerequisite in order to detect Src, which roughly has the same size as the IgG heavy chain (60 kDa and 55 kDa respectively).

### Chromatin immunoprecipitation (ChIP)

ChIP analysis of the Cyclin D1 promoter was performed using starved T47D cells (as described above) treated with 2 nM HRG and/or 10 nM E2 for 30 min. 1 μg of ERα antibody (MC-20, Santa Cruz) or 1 μg of non-specific rabbit IgGs was used for the ChIP, following the protocol described in [22]. The cyclin D1 primers were described in [13]. Detection was performed using qPCR and ERα binding to the promoter was calculated as fold enrichment over IgG.

### Luciferase reporter assay

The luciferase reporter assay was performed as described before [23]. In brief, T47D cells were seeded into 24-well plates (1 × 10^5^ cells/well). The next day, pRL-TK and 3 x ERE-luc vectors were transfected using JetPrime transfection reagent according to manufacturer's instructions (Polyplus Transfection, Illkirch-Graffenstaden, France) using a total of 1 μg plasmid DNA. 4h after transfection, the media was changed to stripped media containing 10 nM E2 and/or 2 nM HRG. 48 h later the luciferase reporter assay was performed [23] and firefly luciferase activity was normalized to renilla luciferase activity.

### Immunofluorescence microscopy

The subcellular localization of ERα and Memo was performed by seeding 300 μl of 3×10^4^ T47D or MCF-7 cells/ml in ibidi 8 well μ-slides (Ibidi, Martinsried, Germany). The cells were starved and treated with 2 nM HRG and/or 10 nM E2 (as described above) for different time points before fixation with 4% formaldehyde. The cells were then permeabilized for 10 min with 0.1% Triton X-100 (Merck) and blocked with 10% horse serum in PBS (ThermoFisher) for 30 min. The cells were stained with 300 nM 4′,6-diamidino-2-phenylindole (DAPI) for 5 min, washed with PBS and then stained for 2h at room temperature for ERα and Memo using ERα antibody (MC-20, Santa Cruz) and Memo antibody (ab156614, Abcam, Cambridge, UK) respectively, both at 1:50 dilution. After washing with PBS, the cells were incubated with secondary Alexa Fluor antibodies (ThermoFisher) (Supplemental Table II) for 1h at room temperature. The cells were washed, mounted using the ibidi-mounting medium (Ibidi), and imaged using an OlympusIX71 microscope (Olympus, Center Valley, PA, USA) and NIS-Elements BR 3.2 Software (Nikon, Tokyo, Japan). Cells overexpressing GFP-tagged proteins were treated the same but without antibody incubations. Quantification of relative percentage nuclear staining was performed using the ImageJ software (NIH Software) using the algorithm: (Integrated density of nuclear fluorescence - (area of interest x mean fluorescence of background)) / (Integrated density of total cell fluorescence - (area of interest x mean fluorescence of background)). The image acquisitions and quantifications were made in a double-blind procedure.

### Construction of ERα-Y537F plasmid and transfections

pEGFP-ERα-C1-GFP (ERα-GFP, WT) was purchased from Addgene (Cambridge, MA, USA) and used as backbone to construct pEGFP-ERα-C1-GFP-Y537F (ERα-Y537F-GFP) using the GeneArt site-directed mutagenesis system (ThermoFisher) following manufacturers protocol. The primers used to introduce the Y537F mutation were 5′-GAA CGTGGTGCCCCTCTTTGACCTGCTGCTGGAGA-3′ (forward) and 5′-TCTC CAGCAGCAGGTCAAAGAGGGGCACCACGTTC-3′ (reverse). The vector was transformed into competent Top10 cells (ThermoFisher), amplified, purified, and sequenced. The pEGFP-ERα-C1-GFP and pEGFP-ERα-C1-GFP-Y537F plasmids were used to transfect T47D NT and Sh5 cells using Lipofectamin 3000 system (ThermoFisher) following manufacturers recommendations. Visualization of ERα-GFP expressing cells was done 2 days after transfection using an OlympusIX71 microscope (Olympus).

### Migration and proliferation assays

The cell migration was measured using 8 μm pore polycarbonate membrane transwell chambers (BD Biosciences, Stockholm, Sweden) coated with 25 μg/ml rat tail collagen (Roche) as described before with some modifications [24]. Cells were starved in 0.5% DCC-treated FCS in phenol-free DMEM for 48h and then seeded at 25 000 cells/well each transwell chamber and let migrate towards vehicle (DMSO), 2 nM HRG, 10 nM E2 for 24h and/or 20 nM 4-OHT. The non-migrated cells were scraped from the top membrane using a cotton swab and the migrated cells were fixed in 4% para-formaldehyde and stained in 0.1% crystal violet (Sigma Aldrich). The cells were imaged using an Axiovert S100 inverted microscope (Carl Zeiss, Oberkochen, Germany). The migrated cells were counted in five different fields in duplicate wells, in at least three independent experiments and expressed as the mean ± S.E.M.

For proliferation assays, 25 000 cells/ml were let to adhere to the culture plate for 4 h in plain medium and then starved in phenol-free DMEM supplemented with 1% DCC-treated FCS for five days in the presence of vehicle (DMSO), 2 nM HRG, 10 nM E2 for 24h and/or 20 nM 4-OHT. Viable cells were counted using Trypan blue exclusion in at least three independent experiments and expressed as the mean ± S.E.M.

### Statistical analysis

The data represent means ±S.D. or ± S.E.M. from three or more independent experiments. Sample sizes were selected on the basis of preliminary experiments to ensure adequate power. After confirming that the data were normally distributed based on Shapiro-Wilk's normality test (Prism v. 6.0, GraphPad Software, La Jolla, CA, USA), statistical significances were determined using an unpaired, two-tailed Student's t-test, assuming unequal variances in single comparisons. For multiple comparisons one-way ANOVA followed by the Tukey's *post-hoc* test (assuming equal variances) or Dunnett's *post-hoc* test (assuming unequal variances) was performed. Differences were considered significant if the p-value was p < 0.05.

## SUPPLEMENTARY MATERIAL FIGURES AND TABLES


