# Supplementary material for: Memo interacts with c-Src to control Estrogen Receptor alpha sub-cellular localization
Source: Oncotarget. 2016 Jul 26;7(35):56170–82. doi: 10.18632/oncotarget.10856 (PMC5302904; doi:10.18632/oncotarget.10856)
Supplement: Supplementary file 1 [file oncotarget-07-56170-s001.pdf]

Memo interacts with c-Src to control Estrogen Receptor alpha sub-cellular localization

Supplementary Material

Supplemental Table I. Primer list

Real-time qPCR primers

Primer	Forward (5'-3')	Reverse (5'-3')
GREB1	GGCTTTTAACACGTGTGGTG	GTGTAGCCTTCGCTCAGCA
PS2	CCCCTGGTGCTTCTATCCTA	GATCCCTGCAGAAGTGTCTAAAA
Cyclin D1	GCTGCGAAGTGGAAACCATC	CCTCCTTCTGCACACATTTGAA
Estrogen Receptor alpha	TTACTGACCAACCTGGCAGA	ATCATGGAGGGTCAAATCCA
18S rRNA	GGACATCTAAGGGCATCACAGACC	TGACTCAACACGGGAAACCTCAC

Supplemental Table II
Antibody reference list

Primary antibodies

Antibody	Species	Company	Cat no./Clone	Citation/reference	Dilution
Memo	Mouse	In-house	1469	(1)	1:3000
Memo	Mouse	Abcam	Ab156614	(2)	1:3000
ERα	Rabbit	Santa-Cruz Biotechnology	MC-20, Sc-542	(3)	1:1000
PY537-ERα	Rabbit	Abcam	Ab192434	(4)	1:800
PS118-ERα	Rabbit	Millipore	05-793 / NL44	(5)	1:800
PS167-ERα	Rabbit	Cell Signaling	5587	(6)	1:800
PY1248-HER2	Rabbit	Cell Signaling	2247	(7)	1:1000
PY418-Src	Rabbit	Life Technologies	44-660G	(8)	1:800
PS473-Akt	Rabbit	Cell Signaling	9271	(9)	1:1000
PT202/Y204-Erk1/2	Rabbit	Cell Signaling	9101	(10)	1:1000
HER2	Rabbit	Santa Cruz	C-18, Sc-284	(11)	1:1000
Src	Rabbit	Santa Cruz	N-16, Sc-19	(12)	1:1000
β-Actin	Mouse	Millipore	MAB1501	(13)	1:10 000

Secondary antibodies

Antibody	Species	Company	Cat no./Clone	Citation/reference	Dilution
Anti-Rabbit HRP-linked	Sheep	GE Healthcare	NA934V	(13)	1:10 000
Anti-Mouse HRP-linked	Sheep	GE Healthcare	NA931V	(13)	1:10 000
Alexa Fluor 568 Anti-mouse	Goat	Life Technologies	A11031	(14)	1:200
Alexa Fluor 488 Anti-Rabbit	Goat	Life Technologies	A11034	(14)	1:200

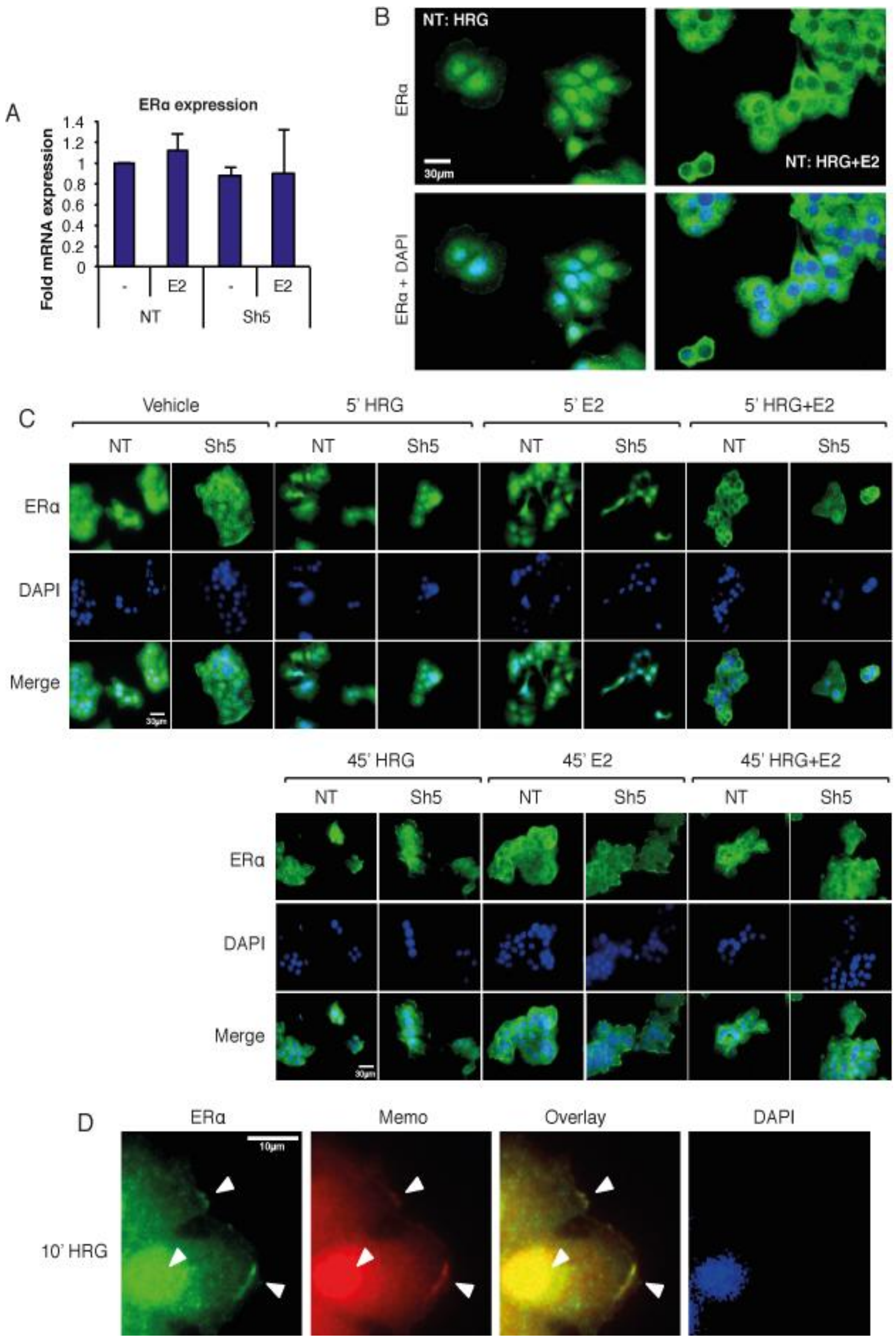

Supplemental Figure 1. Expression and localization of ERα. (A) Relative ERα mRNA expression in NT and Sh5 T47D cells upon 24h E2 treatment (n=3). (B) Magnification of ERα Immunofluorescence (IF) in T47D cells depicted in figure 1G upon HRG and HRG+E2 treatment. (C) IF analysis of ERα

localization upon treatment with DMSO (vehicle), 2 nM HRG, and/or 10 nM E2 for 5 min (short treatment) or 45 min (long treatment). Nuclei were stained with DAPI. 40x magnification, scale bar: 30 µM (n=3). (D) ERα co-localization with Memo upon 10 min 2 nM HRG treatment. Arrows show co-localization at the membrane and the nucleus, scale bar: 30 µM. The data shown in (A) represent means and error bars represent S.D.

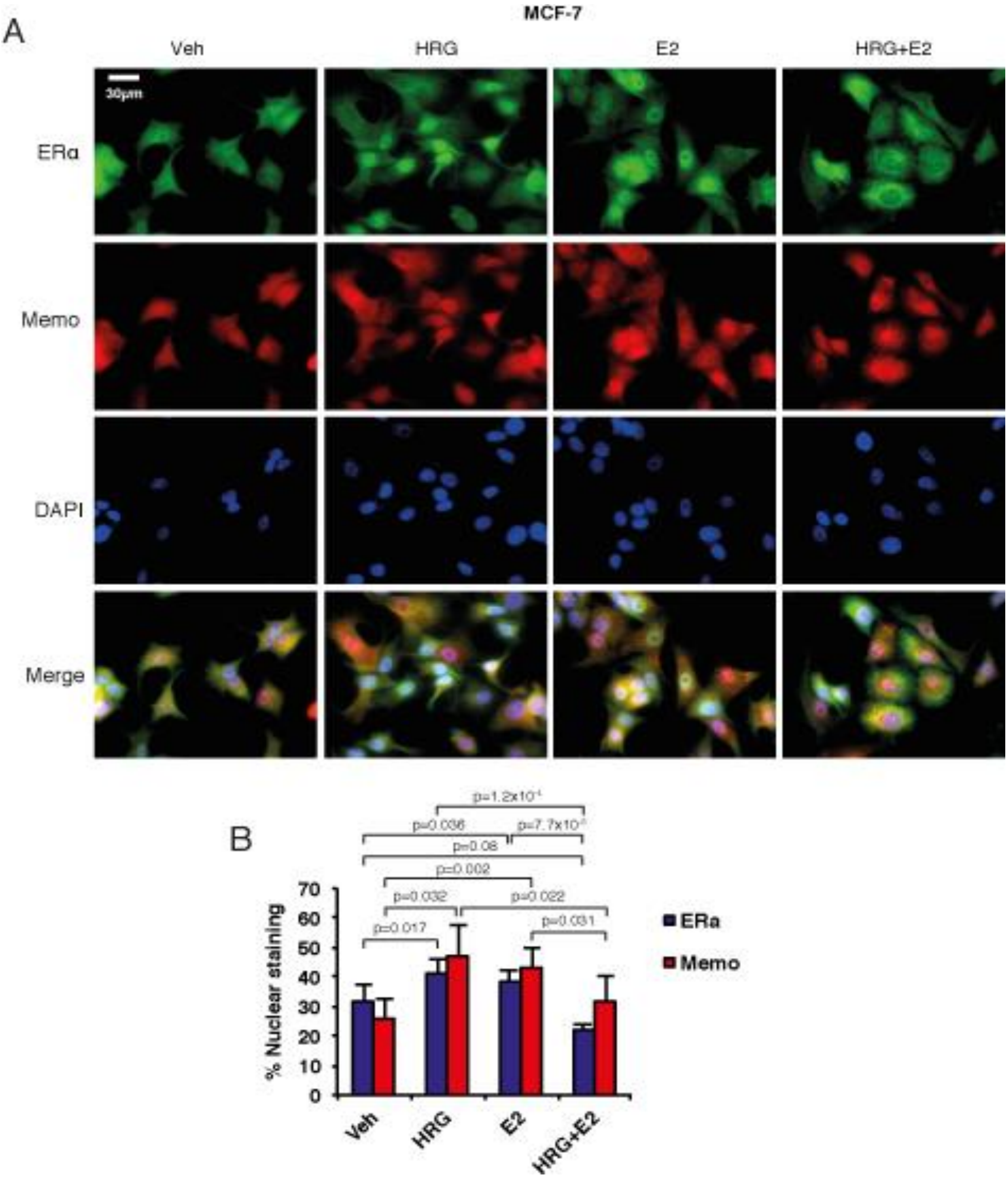

Supplemental figure 2. Sub-cellular localization of ERα and Memo in MCF-7 breast cancer cells. (A) Immunofluorescence (IF) analysis of ERα localization upon treatment with DMSO (vehicle), 2 nM HRG, and/or 10 nM E2 for 10 min. Nuclei were stained with DAPI. 40x magnification, scale bar: 30 µM. (B) Quantification of nuclear ERα IF intensity as percentage of total ERα IF intensity (n=6). The data shown in (B) represent means and error bars

represent standard deviation (SD), *P* values were determined using one-way ANOVA.

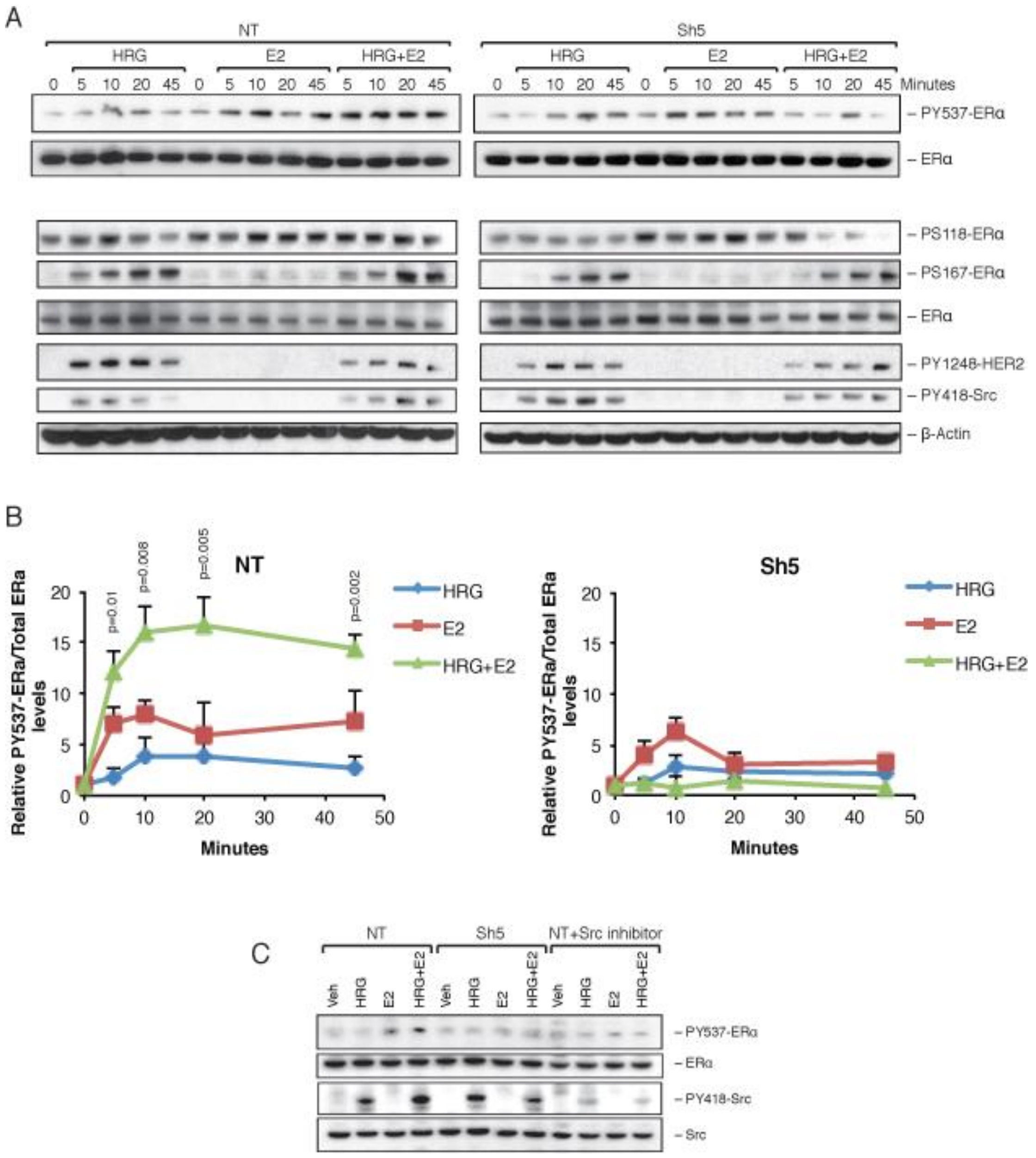

Supplemental Figure 3. Time-course analysis of ERα, HER2 and Src phosphorylation in starved NT and Sh5 T47D cells. (A) Western blot analysis of ERα, HER2 and Src phosphorylation after the indicated time of HRG and/or E2 treatment. (B) Quantification of relative PY537-ERα levels observed in (A) (n=3). (C) Western blot analysis of PY537-ERα and PY418-Src in starved T47D cells treated for 10 minutes with 2 nM HRG, 10 nM E2, DMSO (Veh),

with or without 500 nM Src inhibitor1. The data shown in (B) represent means and error bars represent standard deviation (SD), *P* values were determined using Student's t-test.

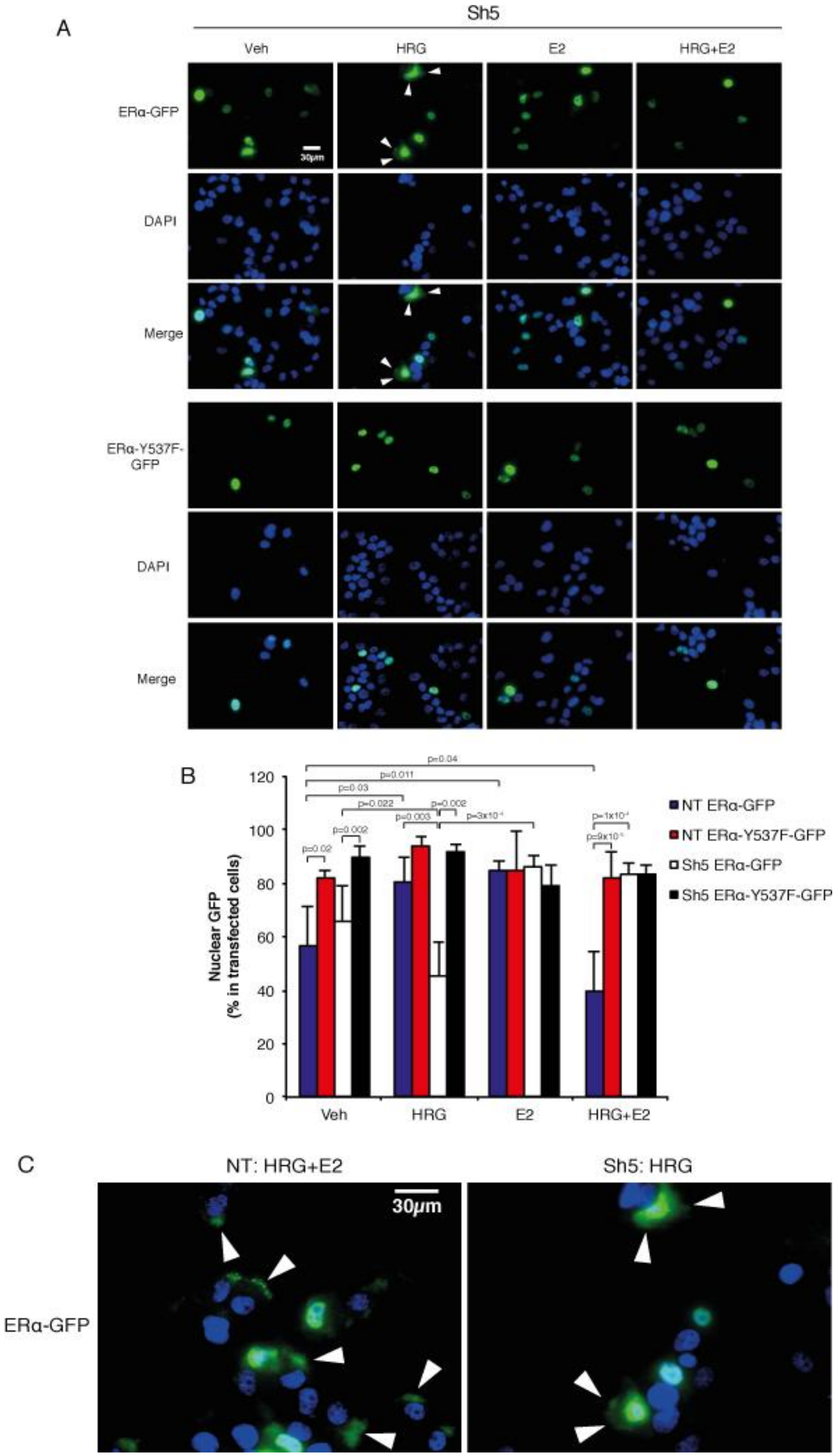
A
Sh5
Veh
HRG
E2
HRG+E2
ERα-GFP
30μm
DAPI
Merge
ERα-Y537F-GFP
DAPI
Merge
B
Nuclear GFP (% in transfected cells)
0
20
40
60
80
100
120
Veh
HRG
E2
HRG+E2
NT ERα-GFP
NT ERα-Y537F-GFP
Sh5 ERα-GFP
Sh5 ERα-Y537F-GFP
p=0.04
p=0.011
p=0.03
p=0.022
p=0.002
p=0.003
p=0.002
p=0.02
C
NT: HRG+E2
Sh5: HRG
30μm
ERα-GFP

Supplemental Figure 4. Localization of ERα-GFP in T47D NT and Sh5 cells. (A) Immunofluorescence (IF) analysis of ERα-GFP localization upon treatment with DMSO (vehicle), 2 nM HRG, and/or 10 nM E2 for 10 min. Nuclei were stained with DAPI. 40x magnification, scale bar: 30 µM. Arrows show extra-nuclear ERα-GFP localization. (B) Quantification of nuclear ERα-GFP IF intensity as percentage of total ERα IF intensity (n=6). (C) Magnified pictures of (A) showing extra-nuclear ERα-GFP (arrows). The data shown in (B) represent means and error bars represent standard deviation (SD), *P* values were determined using one-way ANOVA.

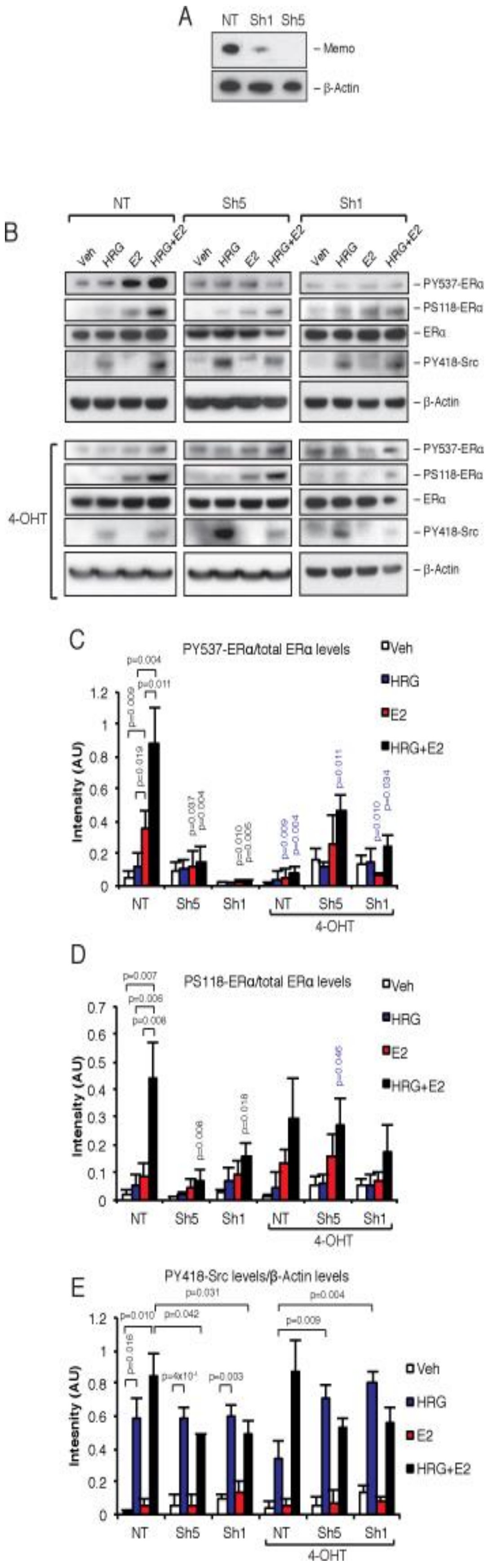
A
NT Sh1 Sh5
Memo
β-Actin
B
NT
Sh5
Sh1
Veh HRG E2 HRG+E2
PY537-ERα
PS118-ERα
ERα
PY418-Src
β-Actin
4-OHT
C
PY537-ERα/total ERα levels
Intensity (AU)
Veh
HRG
E2
HRG+E2
4-OHT
D
PS118-ERα/total ERα levels
Intensity (AU)
E
PY418-Src levels/β-Actin levels
Intesnity (AU)

Supplemental Figure 5. Comparison of T47D NT and Sh5 cells to Sh1 cells. (A) Western blot analysis of Memo levels in T47D NT, Sh1, and Sh5 cells. (B) Western blot analysis of PY537-ERα, PS118-ERα, and PY418-Src levels in starved T47D cells treated with 2 nM HRG, 10 nM E2, DMSO (Veh), and/or 20 nM 4-OHT for 10 min. (C) Quantification of PY537-ERα levels relative to total ERα levels (AU, arbitrary units) (n=3). (D) Quantification of PS118-ERα levels relative to total ERα levels (AU, arbitrary units) (n=3). (E) Quantification of PY418-Src levels relative to β-Actin levels (AU, arbitrary units) (n=3). The data shown in (C – E) represent means and error bars represent S.D. Significance levels written in blue show the significance between 4-OHT treatment and the respective treatment without 4-OHT. *P* values were determined using Student's t-test one-way ANOVA.

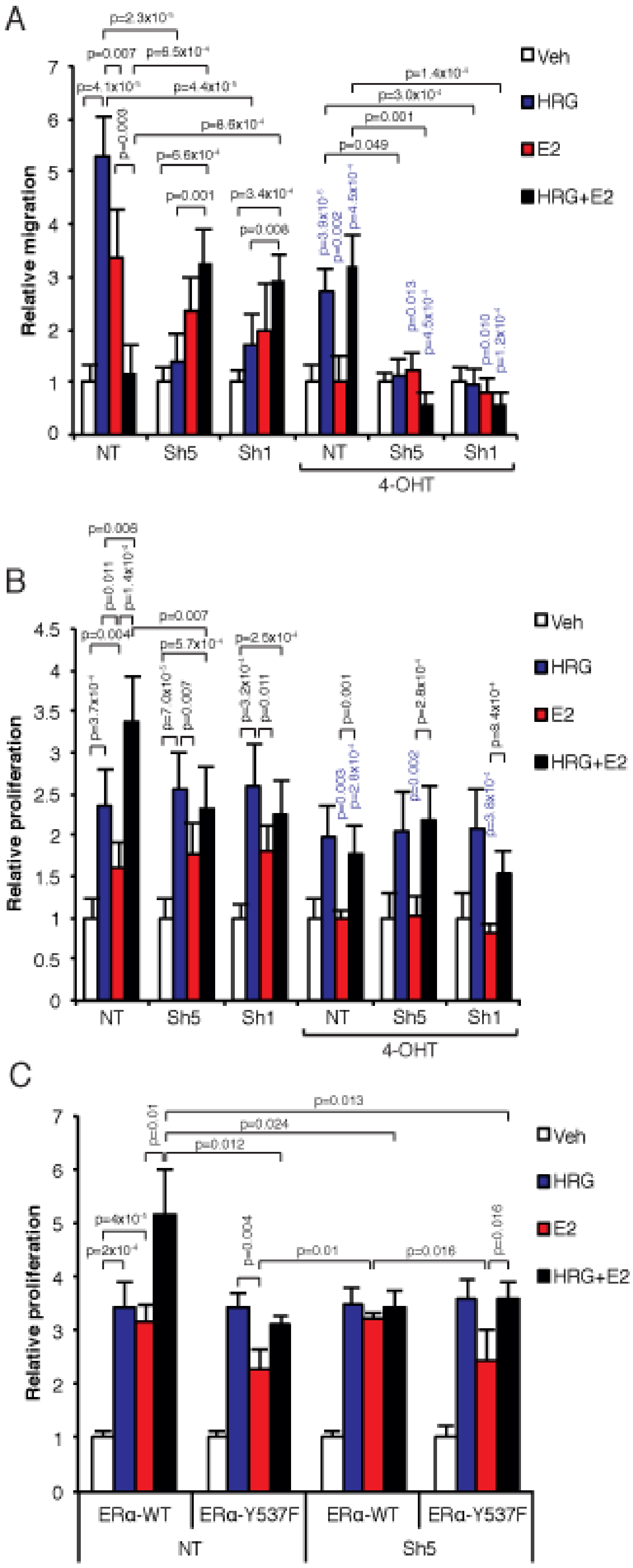
A
Relative migration
Veh
HRG
E2
HRG+E2
NT
Sh5
Sh1
NT
Sh5
Sh1
4-OHT
B
Relative proliferation
Veh
HRG
E2
HRG+E2
NT
Sh5
Sh1
NT
Sh5
Sh1
4-OHT
C
Relative proliferation
Veh
HRG
E2
HRG+E2
p=0.013
p=0.024
p=0.012
p=0.01
p=4x10⁻⁵
p=2x10⁻⁴
p=0.004
p=0.01
p=0.018
p=0.016
ERα-WT
ERα-Y537F
ERα-WT
ERα-Y537F
NT
Sh5

Supplemental Figure 6. Comparison of migration and proliferation of T47D NT and Sh5 cells to Sh1 cells, as well as to ERα-Y537F cells. (A) Migration assay. Starved T47D cells were seeded into the upper transwell chamber. The lower wells contained phenol-free DMEM supplemented with 0.5% DCC-treated FCS and 2 nM HRG, 10 nM E2, DMSO (Veh), and/or 20 nM 4-OHT. After 24 h the migrated cells were fixed, stained and counted. The migration is expressed relative to respective DMSO treated cells (n=5). (B) Proliferation assay. T47D cells were starved for 5 days in phenol-free DMEM supplemented with 0.5% DCC-treated FCS, and in the presence of 2 nM HRG, 10 nM E2, DMSO (Veh), and/or 20 nM 4-OHT. Viable cells were counted and the proliferation change was assessed relative to respective DMSO treated cells (n=5). (C) Proliferation assay of T47D cells overexpressing ERα-WT-GFP or ERα-Y537F-GFP were starved for 5 days in phenol-free DMEM supplemented with 0.5% DCC-treated FCS, and in the presence of 2 nM HRG, 10 nM E2, and DMSO (Veh). Viable cells were counted and the proliferation change was assessed relative to respective DMSO treated cells (n=3). The data represent means and error bars represent S.E.M. Significance levels written in blue show the significance between 4-OHT treatment and the respective treatment without 4-OHT. *P* values were determined using Student's t-test or one-way ANOVA.

Supplemental Table I. Primer list

Real-time qPCR primers

Primer	Forward (5'-3')	Reverse (5'-3')
GREB1	GGCTTTTAACACGTGTGGTG	GTGTAGCCTTCGCTCAGCA
PS2	CCCCTGGTGCTTCTATCCTA	GATCCCTGCAGAAGTGTCTAAAA
Cyclin D1	GCTGCGAAGTGGAAACCATC	CCTCCTTCTGCACACATTTGAA
Estrogen Receptor alpha	TTACTGACCAACCTGGCAGA	ATCATGGAGGGTCAAATCCA
18S rRNA	GGACATCTAAGGGCATCACAGACC	TGACTCAACACGGGAAACCTCAC

Supplemental Table II
Antibody reference list

Primary antibodies

Antibody	Species	Company	Cat no./Clone	Citation/reference	Dilution
Memo	Mouse	In-house	1469	(1)	1:3000
Memo	Mouse	Abcam	Ab156614	(2)	1:3000
ERα	Rabbit	Santa-Cruz Biotechnology	MC-20, Sc-542	(3)	1:1000
PY537-ERα	Rabbit	Abcam	Ab192434	(4)	1:800
PS118-ERα	Rabbit	Millipore	05-793 / NL44	(5)	1:800
PS167-ERα	Rabbit	Cell Signaling	5587	(6)	1:800
PY1248-HER2	Rabbit	Cell Signaling	2247	(7)	1:1000
PY418-Src	Rabbit	Life Technologies	44-660G	(8)	1:800
PS473-Akt	Rabbit	Cell Signaling	9271	(9)	1:1000
PT202/Y204-Erk1/2	Rabbit	Cell Signaling	9101	(10)	1:1000
HER2	Rabbit	Santa Cruz	C-18, Sc-284	(11)	1:1000
Src	Rabbit	Santa Cruz	N-16, Sc-19	(12)	1:1000
β-Actin	Mouse	Millipore	MAB1501	(13)	1:10 000

Secondary antibodies

Antibody	Species	Company	Cat no./Clone	Citation/reference	Dilution
Anti-Rabbit HRP-linked	Sheep	GE Healthcare	NA934V	(13)	1:10 000
Anti-Mouse HRP-linked	Sheep	GE Healthcare	NA931V	(13)	1:10 000
Alexa Fluor 568 Anti-mouse	Goat	Life Technologies	A11031	(14)	1:200
Alexa Fluor 488 Anti-Rabbit	Goat	Life Technologies	A11034	(14)	1:200

SUPPLEMENTARY REFERENCES

1. Marone R, Hess D, Dankort D, Muller WJ, Hynes NE, Badache A. Memo mediates ErbB2-driven cell motility. Nature cell biology. 2004 Jun;6(6):515-22. PubMed PMID: 15156151.
2. Jiang K, Yang Z, Cheng L, Wang S, Ning K, Zhou L, et al. Mediator of ERBB2-driven cell motility (MEMO) promotes extranuclear estrogen receptor signaling involving the growth factor receptors IGF1R and ERBB2. The Journal of biological chemistry. 2013 Aug 23;288(34):24590-9. PubMed PMID: 23861392. Pubmed Central PMCID: 3750157.
3. Adlanmerini M, Solinhac R, Abot A, Fabre A, Raymond-Letron I, Guihot AL, et al. Mutation of the palmitoylation site of estrogen receptor alpha in vivo reveals tissue-specific roles for membrane versus nuclear actions. Proceedings of the National Academy of Sciences of the United States of America. 2014 Jan 14;111(2):E283-90. PubMed PMID: 24371309. Pubmed Central PMCID: 3896153.
4. Yoshimaru T, Komatsu M, Matsuo T, Chen YA, Murakami Y, Mizuguchi K, et al. Targeting BIG3-PHB2 interaction to overcome tamoxifen resistance in breast cancer cells. Nature communications. 2013;4:2443. PubMed PMID: 24051437. Pubmed Central PMCID: 3791465.
5. Chen D, Washbrook E, Sarwar N, Bates GJ, Pace PE, Thirunuvakkarasu V, et al. Phosphorylation of human estrogen receptor alpha at serine 118 by two distinct signal transduction pathways revealed by phosphorylation-specific antisera. Oncogene. 2002 Jul 25;21(32):4921-31. PubMed PMID: 12118371.
6. Campbell RA, Bhat-Nakshatri P, Patel NM, Constantinidou D, Ali S, Nakshatri H. Phosphatidylinositol 3-kinase/AKT-mediated activation of estrogen receptor alpha: a new model for anti-estrogen resistance. The Journal of biological chemistry. 2001 Mar 30;276(13):9817-24. PubMed PMID: 11139588.
7. Wulfkuhle JD, Berg D, Wolff C, Langer R, Tran K, Illi J, et al. Molecular analysis of HER2 signaling in human breast cancer by functional protein pathway activation mapping. Clinical cancer research : an official journal of the American Association for Cancer Research. 2012 Dec 1;18(23):6426-35. PubMed PMID: 23045247.
8. Sekimoto H, Eipper-Mains J, Pond-Tor S, Boney CM. (alpha)v(beta)3 integrins and Pyk2 mediate insulin-like growth factor I activation of Src and mitogen-activated protein kinase in 3T3-L1 cells. Molecular endocrinology. 2005 Jul;19(7):1859-67. PubMed PMID: 15761030.
9. Hart JR, Vogt PK. Phosphorylation of AKT: a mutational analysis. Oncotarget. 2011 Jun;2(6):467-76. PubMed PMID: 21670491. Pubmed Central PMCID: 3139455.
10. Balko JM, Cook RS, Vaught DB, Kuba MG, Miller TW, Bhola NE, et al. Profiling of residual breast cancers after neoadjuvant chemotherapy identifies DUSP4 deficiency as a mechanism of drug resistance. Nature medicine. 2012 Jul;18(7):1052-9. PubMed PMID: 22683778. Pubmed Central PMCID: 3693569.
11. Gouttenoire EA, Lupo V, Calpena E, Bartesaghi L, Schupfer F, Medard JJ, et al. Sh3tc2 deficiency affects neuregulin-1/ErbB signaling. Glia. 2013 Jul;61(7):1041-51. PubMed PMID: 23553667.

12. Cattaneo F, Iaccio A, Guerra G, Montagnani S, Ammendola R. NADPH-oxidase-dependent reactive oxygen species mediate EGFR transactivation by FPRL1 in WKYMVm-stimulated human lung cancer cells. Free radical biology & medicine. 2011 Sep 15;51(6):1126-36. PubMed PMID: 21708247.
13. MacDonald G, Nalvarte I, Smirnova T, Vecchi M, Aceto N, Dolemeyer A, et al. Memo is a copper-dependent redox protein with an essential role in migration and metastasis. Science signaling. 2014;7(329):ra56. PubMed PMID: 24917593.
14. Hoshiba T, Nemoto E, Sato K, Orui T, Otaki T, Yoshihiro A, et al. Regulation of the Contribution of Integrin to Cell Attachment on Poly(2-Methoxyethyl Acrylate) (PMEA) Analogous Polymers for Attachment-Based Cell Enrichment. PloS one. 2015;10(8):e0136066. PubMed PMID: 26288362. Pubmed Central PMCID: 4545787.
